# Household washing agents and insecticide-treated net bioefficacy: evidence from a rapid review and laboratory evaluation

**DOI:** 10.5281/zenodo.22898431

**Published:** 2026-09-22

**Authors:** Emmanuel Mbuba, Nolvin John Mvungi, Johnson Kyeba Swai, Dickson Kobe, Charles C. Mgaya, Edith Peter Madumla, Edgar Mtaki Mbeyela, Ummi Abdul Kibondo, Jason Moore, Sarah Jane Moore

**Affiliations:** 1 Vector Control Product Testing Unit, Environmental Health and Ecological Sciences Department, Ifakara Health Institute, P.O. Box 74, Bagamoyo, United Republic of Tanzania.; 2 Swiss Tropical and Public Health Institute, Kreuzstrasse 2, CH-4123 Allschwil, Switzerland.; 3 Universität Basel, Petersplatz 1, P.O. Box CH-4001 Basel, Switzerland.; 4 Nelson Mandela African Institution of Science and Technology, School of Life Sciences and Bioengineering, P.O. Box 447, Tengeru, Arusha, United Republic of Tanzania.

## Abstract

**Introduction:**

Household washing of insecticide-treated nets (ITNs) influences their insecticidal durability. The World Health Organization (WHO) recommends the Collaborative International Pesticides Analytical Council (CIPAC) washing agent for standardised laboratory evaluation of ITNs. However, evidence on whether CIPAC reflects household washing practices remains limited. This study synthesised evidence on household ITN washing agents used across Africa and compared mosquito mortality outcomes after laboratory washing with agents commonly used in Tanzania versus CIPAC.

**Materials and Methods:**

A rapid review of studies reporting ITN washing practices in Africa was conducted to identify commonly used household washing agents. An exploratory laboratory study was then performed using an alpha-cypermethrin-incorporated ITN washed with Jamaa®, Family®, OMO®, water only, or CIPAC washing agent across 0–20 wash cycles. Bioefficacy was assessed using WHO cone bioassays against pyrethroid-susceptible An. gambiae and resistant An. arabiensis. Binomial logistic regression assessed the effect of washing agent on mortality, adjusting for wash number.

**Results:**

Thirty-four studies (30,887 household responses) were included in the rapid review. Bar soaps accounted for 59.0% of reported household washing agents. Mortality did not differ significantly between household washing agents and CIPAC for either mosquito strain (all p > 0.05). Water-only washing resulted in significantly higher mortality than CIPAC among susceptible (OR = 60.1, 95% CI: 35.1–103.1; p < 0.001) and resistant mosquitoes (OR = 2.0, 95% CI: 1.4–2.8; p < 0.001). Mortality declined significantly with increasing wash number regardless of washing agent.

**Conclusions:**

Bar soaps were the most frequently reported agents used for ITN washing across the settings in Africa included in the review. No significant differences in mosquito mortality were observed between household washing agents and CIPAC in this exploratory laboratory study. These data provide preliminary support for CIPAC as a proxy for household soap where feasible, but further confirmatory studies with a wider range of ITNs and settings are needed.

## Introduction

Insecticide-treated nets (ITNs) are one of the most effective and widely deployed malaria prevention tools in endemic countries. Since their introduction in 2004, over 2.8 billion ITNs have been distributed across Africa, substantially reducing malaria morbidity and mortality [1]. However, the long-term effectiveness of ITNs under operational conditions is influenced by household use practices, particularly washing and drying practices [2], which accelerates insecticide loss and contributes to declining chemical durability and bioefficacy over time [3–6].

The insecticidal durability of ITNs under household use is affected by several factors, including washing frequency, washing methods, drying practices, and the type of washing agent used [2,5,7]. Laundry washing agents are broadly classified into two groups: soaps and detergents. Although both function as surfactants that remove dirt and oils they differ considerably. Soaps are produced from natural fats or oils through reaction with an alkali (saponification) and contain mainly sodium palmate or sodium stearate. Soaps work best in soft water because they may form insoluble precipitates in the presence of calcium and magnesium ions. Detergents may also contain enzymes, optical brighteners and fragrances, while soaps may contain fragrances and other additives [8]. These differences in chemical composition and cleaning performance may influence the rate at which insecticides are removed from ITNs during washing [3-5].

The WHO recommends washing ITNs gently with mild soap and water because harsh detergents may accelerate insecticide depletion and compromise chemical durability [5]. To standardise the evaluation of insecticidal durability, WHO laboratory washing procedures use the CIPAC washing agent or equivalent e.g., Savon de Marseille soap [9]. The CIPAC washing agent contains sodium oleate and polyoxyethylene glycol monostearate, while Savon de Marseille consists primarily of sodium salts of natural fatty acids derived from vegetable (usually palm) oils. The CIPAC washing agent was selected to provide a standardised and reproducible washing medium for evaluating the wash resistance of ITNs under laboratory conditions. Its defined composition enables consistent comparison of ITN products across studies, laboratories, and testing facilities. However, its components may be difficult to obtain in sub-Saharan Africa and need to be carefully stored to prevent oxidation, particularly oleic acid. A recent laboratory suggested that, for polyethylene nets containing incorporated insecticides, detergent choice had limited influence on insecticide retention, with CIPAC and Marseille soaps producing comparable wash outcomes [6].

Long-lasting insecticidal nets (LLINs) are designed to retain insecticidal activity after repeated washing and regular household use [9]. WHO wash-resistance tests use standardised laboratory procedures to ensure reproducibility and comparability across products and testing facilities [9]. However, these procedures may not reflect household washing, where washing agents, water conditions, scrubbing and drying practices vary. In malaria-endemic settings, households use diverse locally available soaps and detergents, selected according to affordability, availability and local customs [10-15]. Yet the agents used to wash ITNs remain poorly documented. Laboratory studies have focused mainly on washing frequency, washing technique and drying practices [2,5], with limited attention to how washing-agent type affects insecticide retention and mosquito mortality. This evidence gap motivated the present study.

Therefore, this study aimed to: (i) synthesise evidence on washing agents used for ITN washing in malaria-endemic settings through a rapid review, and (ii) evaluate the effects of selected commonly used household soaps in Tanzania compared with the CIPAC washing agent on the bioefficacy of an incorporated alpha-cypermethrin ITN, to determine whether standardised washing practice adequately approximates real-world household washing.

## Materials and Methods

### Rapid review search strategy

A structured search of PubMed was conducted to identify studies published between January 2000 and April 2026 using combinations of the following terms: ("insecticide-treated nets" OR "long-lasting insecticidal nets") AND ("washing" OR "soap" OR "detergent" OR "washing agents"). Only publications in the English language were included. Reference lists of eligible studies were screened to identify additional publications. To better capture operational washing practices, evidence from ITN durability studies, unpublished datasets, and programmatic surveys, specifically those conducted under the U.S. President's Malaria Initiative (PMI), were also included.

### Study selection

Studies were included if they reported household ITN washing practices specifying the washing agents used. Studies limited to laboratory evaluations, experimental hut studies, or studies reporting insecticidal efficacy without reporting household washing practices were excluded. Qualitative studies without quantitative information on washing agents and studies that did not specify washing agents were also excluded.

### Data extraction and synthesis

A standardised data extraction form was used to capture key study characteristics, including study country, author and year of publication, sample size (defined as the number of household responses or nets reporting washing agent used), washing agents reported, the proportion of each washing agent used, and the predominant washing agent identified in each study. Due to heterogeneity in study design, reporting approaches, and outcome measures, findings were synthesised narratively. Data reported in individual studies were summarised to identify patterns in washing agent use across malaria-endemic settings.

### Laboratory evaluation

The laboratory evaluation was conducted at the Vector Control Product Testing Unit (VCPTU) of the Ifakara Health Institute (IHI) in Bagamoyo, Tanzania. Bagamoyo District is located in the Coastal Region, approximately 70 km north of Dar es Salaam, at a latitude of 6°37' south and longitude 38°58' east.

#### Mosquito strains

Females of two *Anopheles* strains were used: the pyrethroid-susceptible *An. gambiae sensu stricto* Ifakara strain (colonised since 1996) and the pyrethroid-resistant *An. arabiensis* Kingani strain (colonised since 2005). The resistance status was confirmed using the 1x discriminating concentration of 0.05% alpha-cypermethrin, resulting in 100% mortality in the Ifakara strain and 10% mortality in the Kingani strain. Colonies are maintained under standard insectary conditions (27 ± 2°C, 80± 20% relative humidity, 12:12 h light: dark cycle). Larvae are fed Tetramin® fish food, while adults are maintained on 10% glucose solution and periodically fed blood to sustain colony propagation.

#### Net evaluated and its preparation

The study evaluated an alpha-cypermethrin-incorporated ITN made of mono-filament high-density polyethylene (HDPE) yarn incorporated with 5.8 g/kg ± 25% of alpha-cypermethrin content. Three whole nets were randomly selected, and nine pieces (25 cm × 25 cm) were cut from each net following WHO procedures [9]. From each net, five pieces were selected for washing: one piece per net was allocated to each of the four washing-agent arms (Jamaa®, Family®, OMO®, and CIPAC), and one piece was retained as a water-only control. The study tested three replicate pieces per washing agent per wash day, one from each of the three nets. The remaining four pieces per net were retained as spares and stored at 4°C, but were not used in testing.

#### Soap selection

The laboratory evaluation compared three household washing agents (Jamaa®, Family®, OMO®) because they are among the most widely available and commonly used household washing agents in Tanzania and represent both bar soap and detergent-based formulations. Jamaa® palm oil bar soap and two laundry detergents (Family® and OMO®) were locally purchased and compared with CIPAC washing agent as reference standard washing agent. The commercial washing agents represented specific product samples purchased locally in Tanzania. Their complete formulations and ingredient concentrations were not characterised. The CIPAC washing agent was prepared in-house using sodium oleate from Sigma-Aldrich Co. LLC and polyoxyethylene glycol (25) monostearate from Tokyo Chemical Industry, Ltd, UK. The CIPAC washing agent was prepared by heating polyoxyethylene glycol (25) monostearate to 50°C to reduce viscosity. Sodium oleate (12 g) and polyoxyethylene glycol (25) monostearate (8 g) were dissolved in 80 mL deionised water and heated to 50°C with continuous stirring until a clear, homogeneous solution was obtained. The stock solution was stored at 4-8 °C in a refrigerator for the 20-day duration of the study, in a flask covered with aluminium foil.

#### Net washing procedures

All net samples were washed up to 20 times, with bioefficacy assessed after 0, 1, 2, 3, 5, 10, 15, and 20 washes. Net samples were placed in 500 mL of deionised water containing washing agent, either 1 g of soap / detergent or 2.5 mL of CIPAC. The container was inverted by hand ten times at 180° and then placed in a water bath at 30°C for 10 minutes. The sample was then rinsed twice in fresh deionised water, each rinse involving ten inversions followed by 10 minutes at 30°C. Samples were dried under shade at room temperature (27–30 °C) for 1 hour, and stored at 30°C for 24 hours before the next bioefficacy test and wash. Three net samples were washed per washing agent per wash day. A control sample, washed in deionised water only, followed the same steps but without soap and it was included to distinguish the effects of mechanical washing from soap-mediated insecticide removal. The negative control used to verify assay quality was untreated SafiNet® netting (A to Z Textile Mills, Tanzania).

### Bioefficacy testing

Cone bioassays were conducted on all net samples included in the washing experiment following WHO wash-resistance testing procedures. The same net pieces were tested repeatedly across successive wash cycles [9].

At each wash point (0, 1, 2, 3, 5, 10, 15, and 20 washes), four WHO cones were attached to each net piece, and five sugar-fed, nulliparous female mosquitoes, aged 3–5 days, were introduced into each cone, giving a total of 20 mosquitoes per net piece and 60 mosquitoes per strain in 12 cones per treatment and wash combination. Mosquitoes were exposed to the netting for 3 minutes and then transferred to holding cups supplied with 10% sucrose solution. Knockdown was recorded at 60 minutes (KD60) post-exposure and mortality after a 24-hours holding period. Bioassays were conducted under standard insectary conditions (27 ± 2°C and 80 ± 10% relative humidity). All bioassays for a given wash cycle were performed on the same day before subsequent washing. Both pyrethroid-susceptible *An. gambiae* s.s. (Ifakara strain) and pyrethroid-resistant *An. arabiensis* (Kingani strain) were tested.

### Data management and statistical analysis

Rapid review findings were synthesised and not weighted due to heterogeneity in study reporting. Washing agents were categorised as bar soap, detergent or other, which represented traditional soaps, water-only washing, and unspecified products. The proportion of households reporting each agent was summarised for each region.

For the laboratory evaluation, KD60 and 24-hours corrected mortality were calculated for each mosquito species, soap treatment, and wash number. Mortality was corrected using Abbott's formula as per WHO guidelines [9]. The effect of washing agent on 24-hours mortality was assessed using grouped binomial logistic regression, with the number of dead mosquitoes modelled relative to the total number exposed per cone. Washing agent and wash number were included as categorical main effects, with CIPAC as the reference washing agent. Treating wash number as categorical allowed mortality to vary across wash levels without assuming a linear trend. No interaction term was fitted. Separate models were fitted for each mosquito strain. Results are reported as odds ratios with 95% confidence intervals, with statistical significance set at p < 0.05. Analyses were conducted in Stata version 17.0 (StataCorp, College Station, TX, USA).

## Results

### Rapid review findings

A total of 34 studies were included in the rapid review, comprising 18 peer-reviewed publications, 14 PMI-supported surveys, and two additional datasets from ITN durability studies [16,17], representing a total of 30,887 household responses reporting ITN washing practices (Supplementary Table 1). The included studies covered a broad geographical range across malaria-endemic settings in East and West Africa and demonstrated substantial variation in the washing agents used.

### Washing agents used for ITN across Africa

Bar soap was the predominant washing agent across all included studies, though detergent use varied considerably by setting (Figure 1). In West Africa, bar soap accounted for 58.3% of household washing practices on average, with high use reported in Burkina Faso, Ghana, Liberia, Niger, Nigeria, and Sierra Leone [18–19]. Detergent use in Côte d'Ivoire, urban Cameroon, and Mali, ranged from 37–63% of households [11,13,20]. In East Africa, bar soap predominated at approximately 70.0%, with particularly high use in Uganda (>95%) [22] and Kenya, Rwanda, and Burundi (74–90%) [20,23]. In Tanzania, bar soap remained common (59%), though detergent use ranged from 17–45% depending on the setting [12,23,24]. Detergent use was high in Zanzibar (92%) [26] and parts of Mozambique (70%) [10].

**Figure 1:**
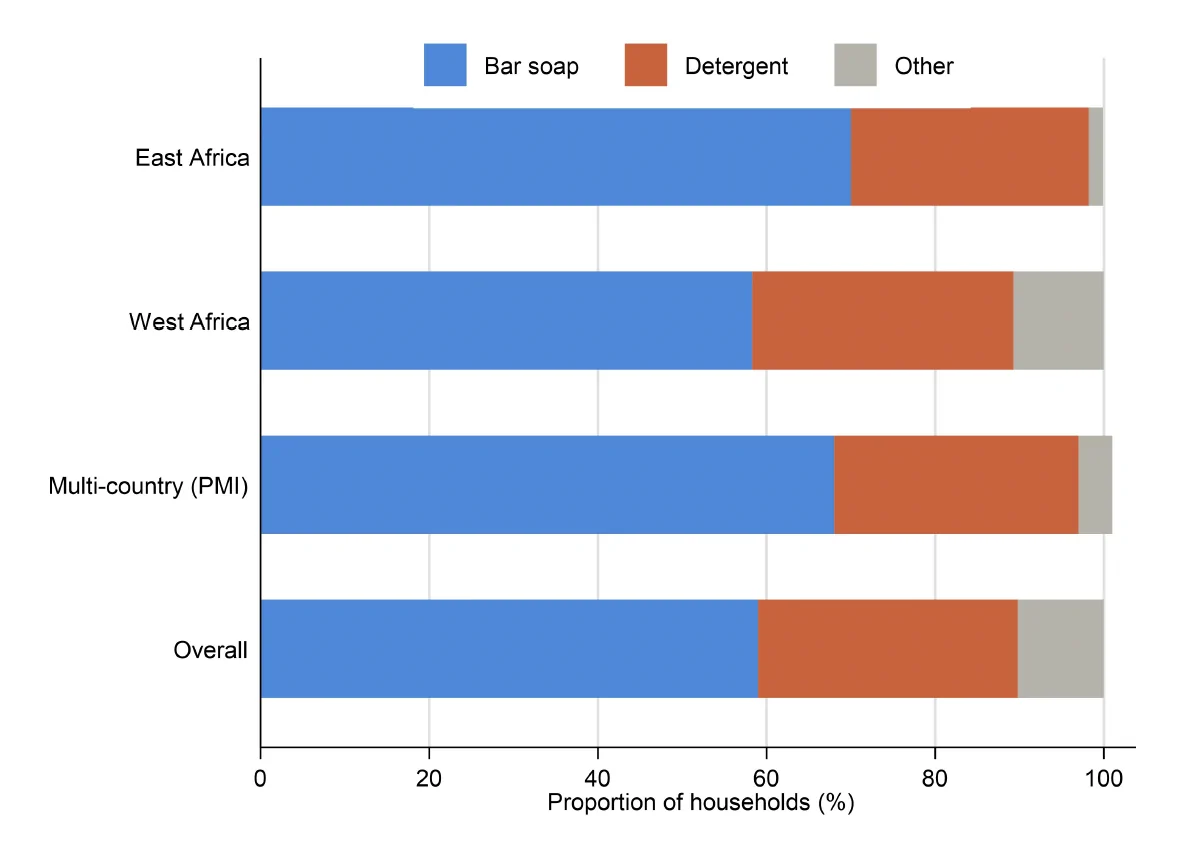
Distribution of washing agents used for ITN washing in Africa (‘Overall’ = 30,877 households). Bars represent the proportion (%) of households reporting use of bar soap, detergent, or other washing agents. "Other" includes traditional soaps, water-only washing, or unspecified agents.

Palm-oil-based bar soaps are compositionally similar to standardised laboratory washing agents; Savon de Marseille and the CIPAC washing agent (Table 1). Most household bar soaps contain sodium salts of fatty acids, such as sodium palmate, sodium palm kernelate, and sodium cocoate, which are chemically comparable to ingredients used in WHO-standardised laboratory washing procedures [9, 26].

**Table 1:** Composition of commonly used household soaps and WHO-recommended washing agents.

Product	Manufacturer	Active ingredients	Common uses
CIPAC washing agent	Not applicable	Sodium oleate; polyoxyethylene glycol	Laboratory studies
Savon de Marseille (unscented)	Multiple manufacturers from the Marseille region	Sodium palmate; sodium palm kernelate; sodium cocoate	Laboratory studies and household use in West Africa
BF, Ideal Plus, Maximousse, Key Soap	Multiple manufacturers	Sodium palmate; sodium palm kernelate; sodium cocoate	Household use in West Africa
Jamaa, White wash, B29, Yellow star, Sunlight bar soap	Produced by multiple manufacturers	Sodium palmate; sodium palm kernelate; sodium cocoate	Household use in East Africa

### Bioefficacy following repeated laboratory washing

KD60 and 24-hours corrected mortality declined progressively with increasing wash cycles across all soap treatments and mosquito species (Figure 2). Against susceptible *An. gambiae s.s.*, the ITN maintained high bioefficacy in early wash cycles, but both KD60 and mortality fell markedly with repeated washing, with mortality declining sharply after 5 washes. Against pyrethroid-resistant *An. arabiensis*, bioefficacy was lower throughout and declined more rapidly, with KD60 approaching near-zero and all soap treatments reaching very low (<20%) mortality after 15-20 wash cycles. Water-only-washed nets retained higher bioefficacy than soap-washed nets at later wash cycles for both species and both endpoints. Water-washed nets continued to kill almost all susceptible mosquitoes. However, repeated washing negatively impacted ITN bioefficacy regardless of soap type. Mortality in untreated nets was below 5% throughout the experiment.

**Figure 2:**
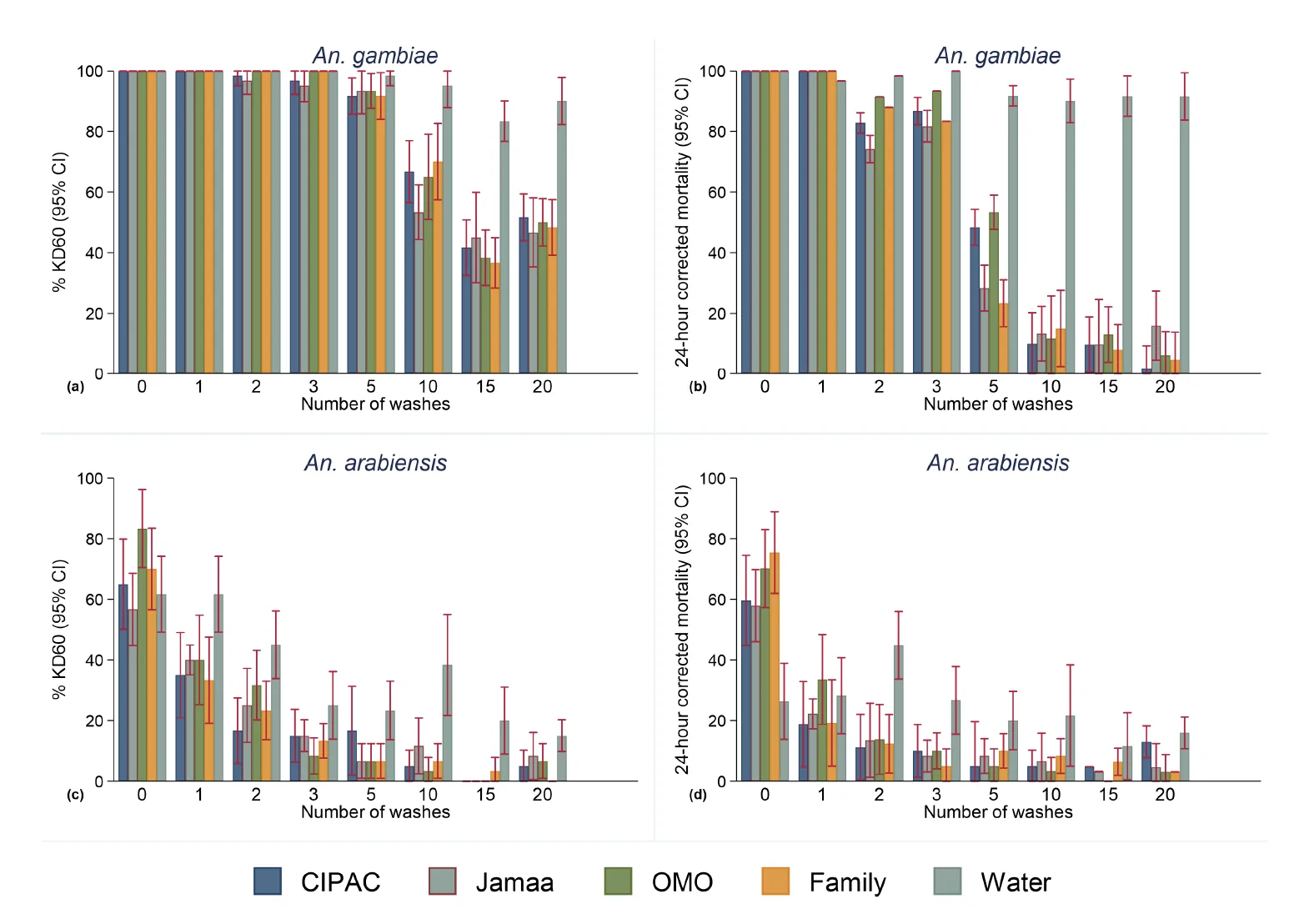
KD60 and 24-hours corrected mortality of susceptible An. gambiae s.s. and pyrethroid-resistant An. arabiensis exposed to nets washed with CIPAC, Jamaa®, OMO®, Family®, or water only across 0–20 wash cycles. Error bars = 95% CI. Absent bars = true zero. A total of 12 WHO cone test replicates (60 mosquitoes) were conducted for each soap type on each experimental day.

### Effect of local soaps on mosquito mortality

After adjusting for the number of washes, there was no evidence that nets washed with Jamaa®, OMO®, or Family® soap differed significantly in 24-hours mosquito mortality from nets washed with the WHO-recommended CIPAC washing agent, for either susceptible *An. gambiae* s.s. or pyrethroid-resistant *An. arabiensis*. Among the susceptible *An. gambiae* s.s., the odds of mortality were similar for Jamaa®, OMO®, and Family® soap relative to CIPAC (Table 2). Likewise, among the pyrethroid-resistant *An. arabiensis*, no significant differences in mortality were observed between CIPAC and any of the locally available soaps evaluated.

**Table 2:** Effect of washing agent on 24-hour mosquito mortality in pyrethroid-susceptible An. gambiae s.s. and pyrethroid-resistant An. arabiensis.

Soap type	*Susceptible An. gambiae* s.s.			*Pyrethroid-resistant An. arabiensis*		
	OR*	95% CI	p-value	OR	95% CI	p-value
CIPAC	Ref.	-	-	-	-	-
Jamaa®	0.82	0.55–1.24	0.349	0.98	0.67–1.43	0.923
OMO®	1.46	0.97–2.18	0.067	1.13	0.78–1.64	0.507
Family®	0.80	0.53–1.21	0.297	1.13	0.78–1.64	0.507
Water only	60.13	35.08–103.07	<0.001	1.97	1.38–2.81	<0.001

* Odds ratios (ORs) and 95% confidence intervals (CIs) were estimated using binomial logistic regression. Estimates are presented relative to the WHO-recommended CIPAC washing agent and adjusted for wash number.

In contrast, nets washed with water only were associated with significantly higher odds of mosquito mortality than CIPAC-washed nets for both strains. For susceptible *An. gambiae* s.s., water-only washing increased the odds of mortality more than sixty-fold (OR = 60.13, 95% CI: 35.08–103.07, p < 0.001), while for pyrethroid-resistant *An. arabiensis*, the odds of mortality were approximately two-fold higher (OR = 1.97, 95% CI: 1.38–2.81, p < 0.001).

## Discussion

This study combined evidence from a rapid review and an exploratory laboratory evaluation to assess household washing agents used for ITN washing and their implications for insecticidal durability assessment. The findings show that bar soaps predominate as the household washing agent of choice across malaria-endemic settings in Africa, and that the specific household washing agents commonly used in Tanzania and evaluated in this study produced mosquito mortality outcomes comparable to those obtained with the WHO-recommended CIPAC washing agent.

The rapid review found bar soap use to be widespread across East and West Africa [18,19,21, 22], while detergent use was more common in certain urban and coastal settings [10,11,13]. Most household bar soaps contain sodium salts of fatty acids, including sodium palmate, sodium palm kernelate, and sodium cocoate, which are compositionally similar to ingredients used in Savon de Marseille soap and the CIPAC washing agent [9]. This compositional overlap supports the relevance of WHO-standardised washing procedures in settings where bar soaps predominate [9]. Nevertheless, considerable variation in household washing agents was observed across settings, with detergent use particularly common in Zanzibar, Mozambique, Côte d’Ivoire, urban Cameroon, and Mali [10,11,13,25].

Interpreting these findings requires considering the factors that govern insecticide loss during washing. The concentration and volume of washing agent, water temperature, agitation, and washing duration together determine the ratio between surface insecticide concentration and the reservoir content remaining after a wash. These parameters must therefore be carefully standardised. The CIPAC washing procedure serves this purpose in the laboratory by providing a defined, controlled washing medium that minimises the heterogeneity present in bar soaps and detergents, allowing consistent comparison of ITN products across studies and testing facilities. Standardised testing remains essential for generating comparable, interpretable measures of insecticidal durability, even though no single test can capture the full diversity of household washing practices. As our findings below indicate, this is justified where the standardised washing agent's biological effect approximates that of the washing agents evaluated in this study [28].

Under the standardised washing procedure used, and after adjustment for wash number, no statistically significant differences in mosquito mortality were detected between nets washed with Jamaa®, Family® or OMO® and those washed with CIPAC, for either pyrethroid-susceptible *An. gambiae* s.s. or pyrethroid-resistant *An. arabiensis*. This finding is specific to the alpha-cypermethrin-incorporated polyethylene ITN and experimental conditions evaluated and should not be interpreted as demonstrating equivalence across washing agents or other ITN products. Similar findings have been reported for polyethylene nets containing incorporated insecticides, where detergent choice had little influence on insecticide retention and washing frequency was identified as the primary determinant of insecticide loss [6]. In contrast, water-only washing retained significantly higher mosquito mortality than soap-based washing, indicating that surfactants contribute to insecticide removal beyond the mechanical effects of washing alone. Water-only washing may therefore result in lower insecticide loss than washing with soap or detergent. This protective effect of water-only washing was far larger for susceptible *An. gambiae* s.s. (OR = 60.1) than for resistant *An. arabiensis* (OR = 2.0), indicating that resistance diminishes the relationship between surface insecticide concentration and mosquito mortality. Regardless of washing agent, repeated washing was associated with substantial declines in mosquito mortality, consistent with previous studies showing that repeated washing accelerates insecticide depletion and reduces ITN bioefficacy over time [2-4]. The consistency of this pattern across both susceptible and resistant mosquito strains indicates that washing frequency, rather than the specific washing agent evaluated, was the principal determinant of declining ITN bioefficacy under the conditions of this study.

No other studies directly comparing CIPAC to household washing agents were found in the literature. Most studies performed for product listing outside of Europe have used Savon de Marseille or a palm-based soap reported as 'Savon de Marseille-like' or simply 'soap' [27,28] even though these soaps may be subject to batch variability. This is likely due to the cost of procuring the CIPAC components and the need to maintain them without contact with air to minimise oxidation.

This study has limitations. The rapid review relied primarily on a single database and included studies with heterogeneous designs, limiting opportunities for quantitative synthesis. Household washing agents were self-reported and therefore subject to recall bias. In addition, the laboratory evaluation was restricted to a single ITN product, made of HDPE, and only a limited number of washing agents, which limits the generalisability of the findings to other net types, fabric material such as polyester ITNs and products containing other insecticides or synergists. Commercial soap and detergents marketed under the same brand may also vary in their formulations and ingredient concentrations across product varieties, markets and manufacturing batches. The findings reported here apply to the formulations and batches tested and may not represent other products marketed under the same brand or washing practices involving intensive rubbing or scrubbing. The insecticide content of the nets was not measured, and further studies with a greater number of products that explore chemical content as well as bioefficacy are warranted. Nevertheless, the combination of evidence from a rapid review and laboratory testing provides complementary insights into household washing practices and their implications for standardised ITN chemical durability assessments.

## Conclusions

Bar soaps were the predominant household washing agents reported for ITN washing across malaria-endemic settings in Africa. Under the standardised washing procedure, mortality did not differ significantly between the three commercial washing agents and CIPAC. However, these findings are specific to the alpha-cypermethrin-incorporated polyethylene ITN under standardised washing practice and may not apply to household washing practices, particularly intensive rubbing, scrubbing or other ITNs products. We recommend retaining CIPAC washing agent for laboratory evaluation of ITNs due to its defined composition that may support reproducibility and comparability across laboratories and ITN products.

## Appendix

**Table 1:** Supplementary Table 1. Summary of washing agents used for ITNs across included community studies, highlighting the predominance of palm-oil–based bar soaps and the context-specific use of detergents in malaria-endemic settings.

Country	Reference	Sample size (n)	Bar soap (%)*	Detergent (%)	Other (%)	Most used
Burundi	PMI	244	74.2	25.8	-	Bar soap
Cameroon	[14]	75	56.5		-	Bar soap
Cameroon	[14]	185		62.4	1.1	Detergent
DRC	[30]	433	72.0	12.0	-	Bar soap
DRC	PMI	165	81.2	18.8	-	Bar soap
Ethiopia	[15]	4,708	54.7	32.2	8.9	Bar soap
Kenya	[23]	1,134	79.2	20.8	-	Bar soap
Kenya	PMI	189	80.4	19.6	-	Bar soap
Madagascar	PMI	413	68.8	31.2	-	Bar soap
Mozambique	[10]	170	18.2	70.0	11.8	Detergent
Mozambique	PMI	886	82.2	17.8	-	Bar soap
Rwanda	PMI	711	89.7	10.3	-	Bar soap
Tanzania	[12]	83	83.0	17.0	-	Bar soap
Tanzania	[25]	1,306	55.2	44.8	-	Bar soap
Tanzania	Mbuba et al. (unpubl.)	486	82.3	17.3	-	Bar soap
Tanzania	[24]	590	57.5	32.0	23.0	Bar soap
Zanzibar (Tanzania)	[26]	634	8.0	92.0	-	Detergent
Zanzibar (Tanzania)	PMI	243	93.0	7.0	-	Bar soap
Uganda	[22]	2,510	99.6	0.4	-	Bar soap
Uganda	[31]	831	95.1	4.9	-	Bar soap
**EAST AFRICA**		**15,996**	**70.0**	**28.2**	**1.7**	
Burkina Faso	PMI	510	77.3	22.8		Bar soap
Cameroon	[21]	186	20.0	41.0	34.0	Detergent
Cameroon	[32]	85	52.9	37.7	9.4	Bar soap
Cote d'Ivoire	[13]	75	24.0	37.3	29.2	Detergent
Cote d'Ivoire	PMI	229	53.3	46.7	-	Bar soap
Ghana	PMI	230	73.9	26.1	-	Bar soap
Liberia	[18]	275	60.0	19.0	21.0	Bar soap
Liberia	PMI	237	62.0	38.0	-	Bar soap
Mali	[11]	29	23.4	46.7	14.8	Detergent
Niger	PMI	196	86.7	13.3	-	Bar soap
Nigeria	[19]	3,649	56.7	42.2	0.3	Bar soap
Nigeria	PMI	358	94.1	5.9	-	Bar soap
Sierra Leone	PMI	261	74.0	26.0	-	Bar soap
**WEST AFRICA**		**6,320**	**58.3**	**31.0**	**10.7**	**Bar soap**
PMI Multi-country (13 nations)	[20]	18,247	68.0	29.0	4.0	Bar soap
**OVERALL**		**30,887**	**59.0**	**30.8**	**10.2**	**Bar soap**

* Percentages represent the proportion (%) of households reporting use of each washing agent. Percentages may exceed 100% where multiple washing agents were reported. In some studies, totals do not sum to 100% due to incomplete or inconsistent reporting in the sources. "-" indicates data not reported. PMI = President's Malaria Initiative. "Other" includes traditional soaps, water-only washing, or unspecified washing agents. Sample size (n) refers to the number of household responses included in each study.
